# Exogenous Application of Methyl Jasmonate and Salicylic Acid Mitigates Drought-Induced Oxidative Damages in French Bean (*Phaseolus vulgaris* L.)

**DOI:** 10.3390/plants10102066

**Published:** 2021-09-30

**Authors:** Mohammed Mohi-Ud-Din, Dipa Talukder, Motiar Rohman, Jalal Uddin Ahmed, S. V. Krishna Jagadish, Tofazzal Islam, Mirza Hasanuzzaman

**Affiliations:** 1 Department of Crop Botany, Bangabandhu Sheikh Mujibur Rahman Agricultural University, Gazipur 1706, Bangladesh; medipaone@gmail.com (D.T.); jahmed06@bsmrau.edu.bd (J.U.A.); 2 Plant Breeding Division, Bangladesh Agricultural Research Institute, Gazipur 1701, Bangladesh; mrahman@bari.gov.bd; 3 Department of Agronomy, Kansas State University, Manhattan, KS 66506, USA; kjagadish@ksu.edu; 4 Institute of Biotechnology and Genetic Engineering (IBGE), Bangabandhu Sheikh Mujibur Rahman Agricultural University, Gazipur 1706, Bangladesh; 5 Department of Agronomy, Faculty of Agriculture, Sher-e-Bangla Agricultural University, Dhaka 1207, Bangladesh

**Keywords:** abiotic stress, antioxidant defense, phytohormones, photosynthesis, pulse crop, water deficit

## Abstract

Drought stress impairs the normal growth and development of plants through various mechanisms including the induction of cellular oxidative stresses. The aim of this study was to evaluate the effect of the exogenous application of methyl jasmonate (MeJA) and salicylic acid (SA) on the growth, physiology, and antioxidant defense system of drought-stressed French bean plants. Application of MeJA (20 μM) or SA (2 mM) alone caused modest reductions in the harmful effects of drought. However, combined application substantially enhanced drought tolerance by improving the physiological activities and antioxidant defense system. The drought-induced generation of O_2_^●−^ and H_2_O_2_, the MDA content, and the LOX activity were significantly lower in leaves when seeds or leaves were pre-treated with a combination of MeJA (10 μM) and SA (1 mM) than with either hormone alone. The combined application of MeJA and SA to drought-stressed plants also significantly increased the activities of the major antioxidant enzymes superoxide dismutase, catalase, peroxidase, glutathione peroxidase, and glutathione-S-transferase as well as the enzymes of the ascorbate–glutathione cycle. Taken together, our results suggest that seed or foliar application of a combination of MeJA and SA restore growth and normal physiological processes by triggering the antioxidant defense system in drought-stressed plants.

## 1. Introduction

French bean (*Phaseolus vulgaris* L.) is one of the world’s most widely cultivated bean species [1]. For example, in Central and South America it accounts for 90% of total bean production [2]. It is a dual-purpose crop that is grown as pulse (grain) and consumed in the immature stage as a tender vegetable [3]. However, bean cultivation in approximately 60% of the regions around the world is affected by drought stress during different periods of crop growth [1,4]. Drought stress decreases plant growth and development by changing plant morphology and triggering variations in a suite of key physiological and biochemical processes [5]. Drought stress is known to increase leaf proline content and decrease leaf chlorophyll content, relative water content, stomatal conductance, cell membrane stability, and maximal efficiency of photosystem II by interrupting the electron transport chain and chloroplast integrity [6]. Drought stress during the bean flowering period decreases the number of pods per plant and the number of seeds per pod [7], thereby reducing productivity. Frequent, longer, and more severe droughts, accompanied by erratic rainfall, are expected in the 21st century across many regions of the world [8,9], including Bangladesh [10], due to the fact of climate change. This predicted increase in episodes of drought stress will constrain crop cultivation and productivity in the future.

Drought-stressed plants experience oxidative damage due to the generation of reactive oxygen species (ROS; singlet oxygen [^1^O_2_], superoxide radical [O_2_^●−^], hydrogen peroxide [H_2_O_2_], and hydroxyl radical [^•^OH]). The ROS-induced damage to biomolecules is one of the major factors that limit plant growth under drought stress [11]. Increased ROS accumulation severely damages cell membrane integrity by accelerating lipid peroxidation [12,13], protein degradation, and nucleic acid damage [14]. Plants can mitigate the toxicity of ROS by activating a dynamic antioxidative defense system comprised of both non-enzymatic and enzymatic constituents [15,16]. The enzymatic components of this antioxidant defense mechanism contain superoxide dismutase (SOD), four enzymes of the ascorbate–glutathione cycle (i.e., ascorbate peroxidase (APX), monodehydroascorbate reductase (MDHAR), dehydroascorbate reductase (DHAR), and glutathione reductase (GR)), catalase (CAT), glutathione peroxidase (GPX), and glutathione *s*-transferase (GST) [15,17]. The non-enzymatic antioxidants are ascorbate (AsA), glutathione (GSH), tocopherol, flavanones, carotenoids, and anthocyanins, among others [16].

Exogenous application of cellular protectants, such as plant hormones, signaling molecules, and trace elements, is a popular approach in research aimed at enhancing abiotic stress tolerance [18,19,20]. For instance, methyl jasmonate (MeJA) and salicylic acid (SA) are the regulatory phytohormones that play a pivotal role in plant signaling responses to environmental cues through the orchestration of a myriad of defensive mechanisms [20,21,22,23]. The exogenous application of these two hormones together to mustard [18,24], grasses [25], jatropha [22], *Verbascum* [26], and maize [27] has been shown to protect plants against abiotic stresses, including drought, by regulating important physiological processes ranging from photosynthesis to nitrogen and proline metabolism and by activating the antioxidant defense system. Previous studies on wheat [28], maize [27], sweet potato [29], and Eureka lemon [30] have suggested that treatment with 5–20 µM MeJA and/or 0.5–2 mM SA effectively enhances the defense signaling routes to mitigate damage due to the abiotic stresses.

A number of reports have confirmed the protective role of MeJA and SA under abiotic stress conditions; however, few investigations have examined the combined application of these plant signaling molecules for mitigation of oxidative damage in plants under stressful conditions. Moreover, to the best of our knowledge, no studies have aimed to elucidate the impact of exogenous MeJA and SA, independently or in combination, on the amelioration of drought-induced oxidative stress in French bean. Recently, Tayyab et al. [27] demonstrated that the combined application of MeJA and SA significantly improved drought tolerance in maize seedlings. Therefore, the goal of this study was to examine how drought-stressed French bean plants respond to exogenous MeJA and SA, applied both separately and in combination, as seed and foliar pre-treatments. The findings from this study provide new mechanistic insights into the coordinated actions of MeJA and SA on the antioxidant defense system in French beans to enhance tolerance against drought-induced oxidative stress. The specific objectives of the study were (i) to determine the effects of exogenous application of MeJA and SA, separately and in combination, on the morpho-physiological traits of French beans and (ii) to evaluate the role of exogenous MeJA and SA and their combined application on the antioxidant defense system of French bean plants exposed to drought stress conditions.

## 2. Materials and Methods

### 2.1. Plant Material, Experimental Conditions, and Treatments

A popular, high yielding, and widely cultivated French bean variety, BARI Jharsheem-1, was used as the experimental material. Seeds were collected from the Vegetable Division, Horticulture Research Center, Bangladesh Agricultural Research Institute, Gazipur, Bangladesh. The experiment was carried out in a dry and cool environment (Table S1).

Methyl jasmonate (CAS #39924-52-2) and salicylic acid (CAS #69-72-7) were purchased from Sigma–Aldrich Chemie GmbH, Germany. Seeds were surface-sterilized with 1% (*v*/*v*) sodium hypochlorite for 10 min and rinsed thoroughly 3 times with sterile distilled water and then soaked in solutions of 20 μM MeJA and 2 mM SA for 18 h prior to sowing, while seeds for the combined application were soaked in an equal volume mixture of 10 μM MeJA and 1 mM SA [27]. Seeds for the control and drought groups were soaked in sterile water. Seeds were then sown in pots (18 cm in diameter × 23 cm in height) containing 13 kg of silt loam soil (sand 26%, silt 50%, and clay 24%), maintained at a full pot capacity of 30.6% volumetric soil water content. The soil was fertilized properly as recommended by Ahmmed et al. [31]. Ten healthy seeds were sown per pot, maintaining uniform spacing in each pot. Seven days after germination, seedlings were thinned to retain five uniform and healthy seedlings in each pot. Fifteen days after germination, the seedlings, which were previously soaked in the hormones, were again treated with MeJA (20 μM), SA (2 mM), and combined (10 μM MeJA + 1 mM SA) solutions as foliar spray. Another set of plants were sprayed with distilled water. Drought stress was imposed a day after the foliar spray by stopping irrigation until the end of the experiment, while the control pots were well watered. The experiment was a completely randomized design (CRD) with five treatments (control, drought (D), drought-stressed plants treated with methyl jasmonate (D + MeJA), salicylic acid (D + SA), and their combination (D + MeJA + SA)) with four replications. 

### 2.2. Pot Capacity and Water Content of the Experimental Soil

The pot capacity of the soil used was measured prior to the start of the experiment gravimetrically using the procedure of Ogbaga et al. [32]. Briefly, pots of completely water-saturated soil were weighed and then dried to a constant weight at 105 °C. The differences between the weights of the water-saturated and oven-dried soils were used to determine the amount of water required to bring the pots to pot capacity, and volumetric water content (%) was determined accordingly. In addition, the water content (%) of the pot soils (at a 15 cm depth) was monitored daily using a digital soil moisture meter (PMS-714, Lutron Electronic Enterprise Co., Ltd., Taiwan, China).

### 2.3. Growth Parameters

At harvest (25 days after foliar application), root length (RL), shoot length (SL), root dry weight (RDW), shoot dry weight (SDW), leaf dry weight (LDW), and total dry weight (TDW) were calculated from 5 plants in each replication. Root and shoot length were determined from the root–shoot junction to the tip of the longest root and shoot, respectively, using a meter scale. Dry weights were weighed after drying the plant samples at 80 °C until a stable weight was reached.

### 2.4. Physiological Parameters

#### 2.4.1. Leaf Chlorophyll and Pigment Content

At harvest, three 3rd fully expanded leaves from each replicate were used to estimate leaf SPAD value (chlorophyll index) and pigment contents. SPAD value was recorded just before the final harvest with a Chlorophyll Meter (Model: SPAD-502, Minolta Co., Ltd., Tokyo, Japan). Then, the leaves were harvested into Ziplock polybags and brought to the laboratory for pigment extraction. Leaf pigments were extracted in 80% (*v*/*v*) acetone and absorbance of the supernatant was determined with a UV-visible spectrophotometer (GENESYS 10S UV-VIS, Thermo Fisher Scientific, Waltham, MA, USA) at 663, 645, and 470 nm for Chl a, Chl b, and carotenoid content, respectively, and calculated according to Arnon [33].

#### 2.4.2. Canopy Temperature Depression

Canopy temperature was measured on alternate days by a hand-held infrared thermometer (Model- MT4, HTC Instruments, Taipei, Taiwan, China; distance–spot ratio, 12:1). An angle of approximately 30° to the horizontal line and a distance of 30 cm from the 3rd fully opened leaf surface was maintained during measurement of the canopy temperature. Canopy temperature depression (CTD) was determined using the procedure of Fischer et al. [34] as ambient temperature minus leaf temperature. CTD was measured from five leaves in each replicate.

#### 2.4.3. Leaf Relative Water Content

Leaf relative water content (LRWC) was estimated following the procedure of Meher et al. [35]. Briefly, 0.5 g leaf sample was immersed in 100 mL of distilled water for 4 h. The weights of turgid leaf samples were then measured and then oven-dried at 80 °C for 48 h. The dry weights of the samples were recorded until a constant weight was achieved. The procedure was repeated thrice for each replicate.

LRWC (%) = [(Fresh weight − Dry weight)/(Turgid weight − Dry weight)] × 100

#### 2.4.4. Cell Membrane Stability

Cell membrane stability (CMS) was determined following the procedure of Sairam et al. [36]. Briefly, in two sets, 30 leaf discs (0.7 cm in diameter) were taken from three completely opened third leaves and put in test tubes containing 10 mL deionized water. One set of 15 leaf discs was incubated at 40 °C for 30 min and the second set at 100 °C in a boiling water bath for 15 min, and then their electrical conductivities, C_1_ and C_2_, respectively, were read with a conductivity meter. The process was repeated thrice for each replicate. CMS was calculated following the equation:

CMS (%) = [1 − (C_1_/C_2_)] × 100

### 2.5. Biochemical Observations

#### 2.5.1. Proline Content

Leaf proline content was measured spectrophotometrically by an acid-ninhydrin method using the procedure outlined by Bates et al. [37]. The proline content was calculated using a standard curve and reported as µmol g^−1^ fresh weight. 

#### 2.5.2. Oxidative Stress Indicators

Generation of superoxide radicals (O_2_^●−^) were measured following the method of Elstner and Heupel [38] with some modifications. Briefly, 0.3 g fresh leaf tissue was homogenized in 3 mL of 65 mM K–P buffer (pH 7.8) and centrifuged at 5000× *g* for 10 min. With 750 µL supernatant, 675 µL K–P buffer and 70 µL of 10 mM hydroxylamine hydrochloride were added, vortexed, and incubated at 25 °C for 20 min. To the mixture, 375 µL of 17 mM sulfanilamide, 37.5 µL α-naphthylamine, and 337.5 µL K–P buffer were added and vortexed. Then, 2.25 mL diethyl ether was added to the mixture, vortexed again, and incubated for 10 min. The absorbance of the upper clear fraction was recorded at 530 nm. The O_2_^●−^ generation was calculated by comparing a standard curve of NaNO_2_^−^.

Fresh leaf tissues (0.5 g) were homogenized in 3 mL of 5% (*w*/*v*) trichloroacetic acid (TCA). After centrifugation at 11,500× *g* for 10 min, the supernatant was used to determine H_2_O_2_ and malondialdehyde (MDA). The H_2_O_2_ content was determined spectrophotometrically according to the procedure of Yang et al. [39] with some modifications. Briefly, 400 µL supernatant was added to 400 mL of 10 mM potassium phosphate buffer (pH 7.0) and 800 mL of 1 M potassium iodide (KI). The reaction was allowed to proceed in the dark for 1 h before measuring the absorbance at 390 nm. The H_2_O_2_ concentration was calculated using the extinction coefficient of 0.28 μM^−1^ cm^−1^. The methods of Heath and Packer [40] and Mohi-Ud-Din et al. [41] were followed for MDA determination. The MDA content was calculated using an extinction coefficient of 155 mM^−1^ cm^−1^ and represented as nmol g^−1^ FW). 

#### 2.5.3. Extraction and Quantitation of Soluble Protein

Fresh leaf tissue (1:2) (*w*/*v*) was extracted in 0.5 M potassium–phosphate (K–P) buffer (pH 7.0) in ice-cold mortar. The extraction buffer included 1 mM ascorbic acid, 1 M KCl, β-mercaptoethanol, and glycerol. The homogenate was centrifuged at 11,500× *g* for 15 min, and the supernatant was used for enzymatic activities assays. Bradford’s [42] rapid quantification method was used to determine the protein content of each enzyme solution.

#### 2.5.4. Assays of Enzymatic Activities

The lipoxygenase (LOX, EC: 1.13.11.12) activity was assayed spectrophotometrically at 234 nm as per Doderer et al. [43] using linoleic acid as a substrate. The activity was calculated using an extinction coefficient of 25,000 M^−1^ cm^−1^. The superoxide dismutase (SOD, EC: 1.15.1.1) activity was assayed based on the inhibition method of Spitz and Oberley [44]. The catalase (CAT, EC: 1.11.1.6) activity was measured at 240 nm according to the method of Noctor et al. [45] and calculated using an extinction coefficient of 39.4 M^−1^ cm^−1^.

The guaiacol peroxidase (POD, EC: 1.11.1.7) activity was quantified as per the description of Castillo et al. [46] at 470 nm after 1 min and calculated considering an extinction coefficient of 26.6 mM^−1^ cm^−1^. The activity of glutathione peroxidase (GPX, EC: 1.11.1.9) was measured following Elia et al. [47] at 340 nm for 1 min. An extinction coefficient of 6.62 mM^−1^ cm^−1^ was used to compute the activity. The glutathione *S*-transferase (GST, EC: 2.5.1.18) activity was measured spectrophotometrically by following the method of Hossain et al. [48] with model substrate 1-chloro-2,4-dinitrobenzene (CDNB) at 340 nm. The activity was calculated using an extinction coefficient of 9.6 mM^−1^ cm^−1^. 

The activities of glutathione reductase (GR, EC: 1.6.4.2), ascorbate peroxidase (APX, EC: 1.11.1.11), monodehydroascorbate reductase (MDHAR, EC: 1.6.5.4), and dehydroascorbate reductase (DHAR, EC: 1.8.5.1) were measured according to Noctor et al. [45] at 340, 290, 340, and 265 nm, respectively. For all enzymes, absorbance changes were observed for 1 min, and extinction coefficients of 6.2 mM^−1^ cm^−1^, 2.8 mM^−1^ cm^−1^, 6.2 mM^−1^ cm^−1^, and 14 mM^−1^ cm^−1^ were used for the quantification of GR, APX, MDHAR, and DHAR, respectively.

### 2.6. Statistical Analysis 

The data were analyzed using Statistix 10 (https://www.statistix.com/) (accessed on 26 May 2021). The least significant difference (LSD) test was used to compare the means at the *p* ≤ 0.05 significance level. R v.4.1.0 for Windows was used to create a heatmap and to perform principal component analysis (PCA) (http://CRAN.R-project.org/) (accessed on 26 May 2021). Trait mean values were normalized and the library pheatmap was adapted to generate heatmap and hierarchical clusters (distance = Euclidean and method = ward.D2) [49]. PCA was performed with the packages ggplot2, factoextra, and FactoMineR [50,51].

## 3. Results

### 3.1. Soil Water Content

**Figure 1:**
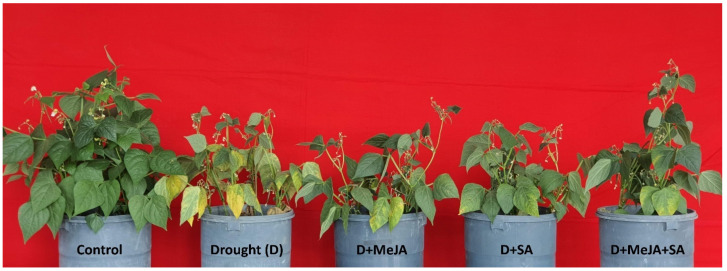
Phenotypic appearance of French bean plants: control, drought (D), and drought-stressed plants treated with methyl jasmonate (D + MeJA), salicylic acid (D + SA), and their combination (D + MeJA + SA) at the time of harvest. Control—plants grown under non-stress, well-irrigated conditions; drought—plants grown with a steady decline in moisture availability; D + MeJA—drought-stressed plants pre-treated (seed and foliar) with 20 µM methyl jasmonate; D + SA— drought-stressed plants pre-treated (seed and foliar) with 2 mM salicylic acid; D + MeJA + SA—drought-stressed plants pre-treated (seed and foliar) with a combination of 10 µM methyl jasmonate and 1 mM salicylic acid.

**Figure 2:**
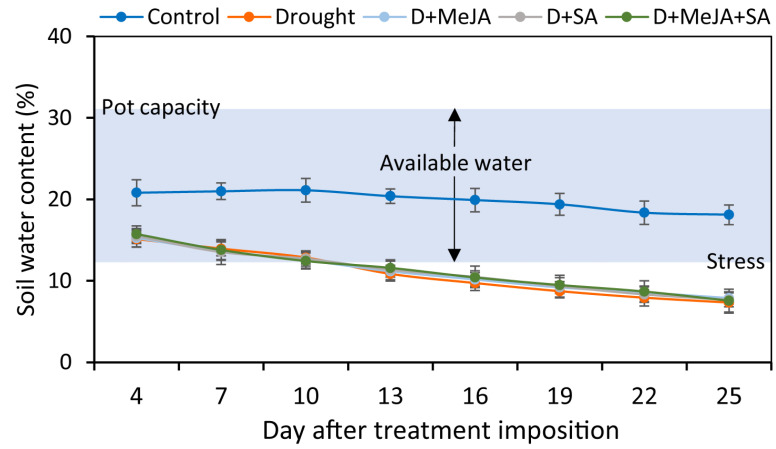
Soil water status of the control, drought (D), and drought-stressed plants treated with methyl jasmonate (D + MeJA), salicylic acid (D + SA), and their combination (D + MeJA + SA) throughout the experimental period. Soil water content was recorded daily for every pot. Each data point indicates the 3-day running average values across four replicates. Vertical bars represent +/− values of the mean. The experimental soil was silt loam (clay:silt:sand = 24:50:26) with a full pot water capacity of 30.6% volumetric soil water content. (Adapted from Pardossi et al. [52]; Weng and Luo [53]).

By the 25th day of treatment, visible effects, including reduced plant height and appearance, increased droopiness, chlorosis, and wilting of leaves, were observed in the French bean plants. Plants were harvested on that day to record observations and conduct chemical analyses (Figure 1). The silt loam used in this study gave a pot capacity of 30.6% volumetric soil water content, determined gravimetrically prior to the start of the experiment (Figure 2). The soil water content was considerably lower for the drought-stressed pots than in the control throughout the experiment (Figure 2). At plant harvest, the soil water contents recorded from the control, drought (D), D + MeJA, D + SA, and D + MeJA + SA treatments were equivalent to 59%, 24%, 26%, 25%, and 25% of pot capacity, respectively (Figure 2), confirming that the symptoms observed in the drought-stressed French bean plants were indicative of water deficit.

### 3.2. MeJA and SA Enhanced Plant Growth

Drought stress decreased the root and shoot lengths of the French bean plants by 5% and 42%, respectively, and the decrease in shoot length was statistically significant (Table 1, Figure S1A). The shoot and root lengths were longer in the drought-stressed plants given hormone treatments than in the drought-stressed plants without hormone treatments (Table 1). Pretreatment of drought-stressed plants with MeJA or SA alone did not result in statistically significant increases in shoot length compared to drought, but the combined (MeJA + SA) application significantly increased the shoot lengths by 20% (Figure S1B). Application of MeJA and SA also improved the plant phenotypic appearance under drought conditions (Figure 2).

Drought stress resulted in a substantial decrease in dry weights. Compared to the unstressed control plants, the root, shoot, leaf, and total dry weights decreased by 52%, 69%, 74%, and 69%, respectively, due to the drought stress (Table 1 and Figure S1A). Exogenous use of MeJA and SA, both alone and in combination, increased the dry weights under the drought-stressed condition, but the increases induced by the combined treatment, except for the root dry weight, were statistically significant. Increases of approximately 35%, 84%, 87%, and 75% in root, shoot, leaf, and total dry weight, respectively, were recorded in the combined treatment compared to drought stress alone (Figure S1B).

### 3.3. Impact of MeJA and SA on Photosynthetic Pigments

The non-destructive chlorophyll index (SPAD) value significantly decreased (20%) under drought stress compared to unstressed control plants (Table 2, Figure S1A). Single and combined applications of MeJA and SA increased the SPAD value of drought-stressed plants compared to untreated drought-stressed plants, but the increase due to the combined treatment (22%) was highly statistically significant (Figure S1B).

Drought stress significantly decreased the leaf levels of chlorophyll a, chlorophyll b, total chlorophyll, and carotenoids by 53%, 51%, 50%, and 48%, respectively, compared to the control (Table 2 and Figure S1A). The exogenous applications of MeJA and SA, separately or in combination, lessened the negative effects of drought stress. However, the increases in pigment levels following application of MeJA or SA alone were not statistically significant. On the contrary, the combined application of MeJA and SA significantly increased chlorophyll a, chlorophyll b, total chlorophyll, and carotenoid contents by 72%, 63%, 64%, and 62%, respectively, compared to the untreated drought-stressed plants (Figure S1B).

### 3.4. MeJA and SA Improved Physiological Traits

**Figure 3:**
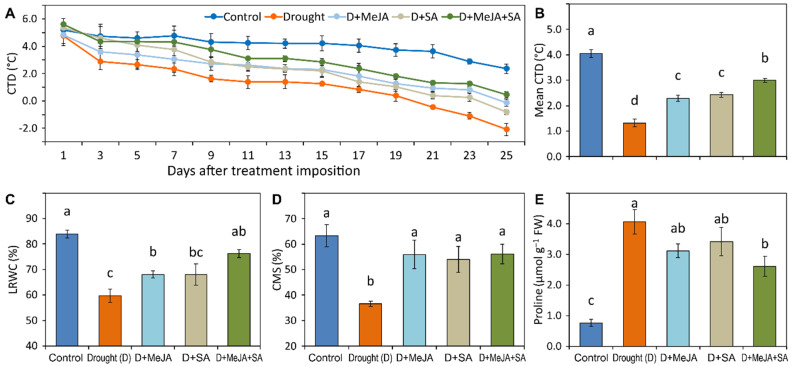
(**A**) Canopy temperature depression (CTD) throughout the experimental period; (**B**) mean CTD; (**C**) leaf relative water content (LRWC); (**D**) cell membrane stability; (**E**) proline content of French bean plants grown under control, drought (D), and drought-stressed plants treated with methyl jasmonate (D + MeJA), salicylic acid (D + SA), and their combination (D + MeJA + SA). Vertical bars represent +/− SE values. Different letter(s) denote a significant difference at *p* ≤ 0.05. FW—Fresh weight. Additional details are shown in Figure 1.

A profound and significant decrease in the mean canopy temperature depression (CTD) was recorded due to the drought stress. The mean CTD decreased by 67% in the drought-stressed plants compared to the control (Figure 3B and Figure S1A), whereas the drought-stressed plants treated with MeJA, SA, or MeJA + SA showed significant increases in mean CTD (73%, 83%, and 127%, respectively) compared to the untreated drought-stressed plants (Figure S1B). Plants treated with the combination of MeJA and SA maintained a greater CTD throughout the course of the experiment compared to plants treated with either MeJA or SA alone (Figure 3A,B). 

The leaf relative water content (LRWC) significantly decreased (29% over the control) by drought stress (Figure 3C and Figure S1A). The drought-stressed plants treated with MeJA and SA and their combination showed increases of 14%, 14%, and 28%, respectively, compared to untreated drought-stressed plants; however, the increase was significant only for the MeJA and the combined hormone treatments (Figure S1B). The cell membrane stability (CMS) of leaves decreased by 42% under drought stress compared to control plants (Figure 3D and Figure S1A). Exogenously applied MeJA and SA mitigated this effect by increasing the CMS by 52% and 47%, respectively, compared to drought-stressed plants (Figure S1B). The protective effect of MeJA + SA was similar to that observed with application of either hormone alone.

Drought stress triggered a profound increase of 436% in the proline content in French bean leaves (Figure 3E and Figure S1A). Application of MeJA, SA, and MeJA + SA to drought-stressed plants lowered the proline content by 23%, 16%, and 36%, respectively, compared to the drought-stressed plants (Figure S1B). 

### 3.5. MeJA and SA Suppressed the Generation of Oxidative Stress Indicators

**Figure 4:**
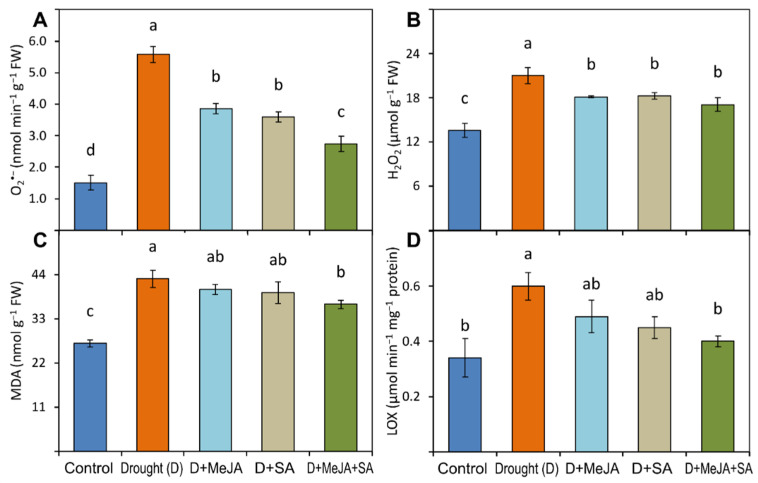
(**A**) superoxide (O_2_^●−^) generation rate; (**B**) hydrogen peroxide content (H_2_O_2_); (**C**) malondialdehyde content (MDA); (**D**) lipoxygenase (LOX) activity of French bean plants grown under control, drought (D), and drought-stressed plants treated with methyl jasmonate (D + MeJA), salicylic acid (D + SA), and their combination (D + MeJA + SA). Vertical bars represent +/− SE values. Different letter(s) denote a significant difference at *p* ≤ 0.05. FW—Fresh weight. Additional details are shown in Figure 1.

Oxidative stress due to the drought stress in French bean plants was determined by measurements of the O_2_^●−^ generation rate, the H_2_O_2_ and MDA levels, and the LOX activity. A sharp increase in O_2_^●−^ generation (270%) was observed in drought-stressed plants compared to unstressed controls (Figure 4A and Figure S2A). Application of MeJA, SA, and MeJA + SA significantly lowered (31%, 36%, and 51%, respectively) O_2_^●−^ generation (Figure 4A and Figure S2B). The H_2_O_2_ levels also significantly increased by 55% in drought-stressed plants compared to the unstressed controls (Figure 4B and Figure S2A). Treatment with MeJA, SA, and MeJA + SA lowered the H_2_O_2_ levels by 14%, 13%, and 19%, respectively, compared to untreated drought-stressed plants (Figure S2B). 

Compared to unstressed controls, the levels of MDA and the LOX activity increased by 60% and 76%, respectively, due to the drought stress (Figure 4C,D and Figure S2A). However, MeJA, SA, and MeJA + SA treatments restrained the production of MDA and the LOX activity in drought-stressed plants. Combined MeJA + SA reduced MDA content and LOX activity by 15% and 33%, respectively.

### 3.6. Antioxidant Enzyme Activities and MeJA and SA Pre-Treatment

**Figure 5:**
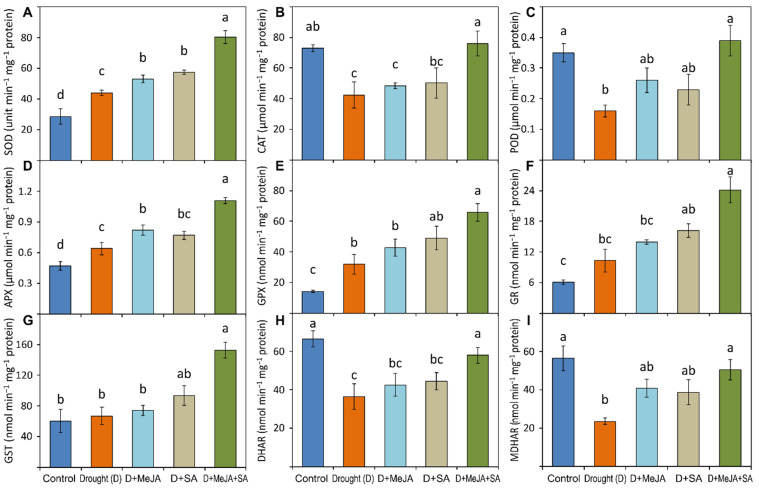
Specific activity of (**A**) superoxide dismutase (SOD); (**B**) catalase (CAT); (**C**) peroxidase (POD); (**D**) ascorbate peroxidase (APX); (**E**) glutathione peroxidase (GPX); (**F**) glutathione reductase (GR); (**G**) glutathione-S-transferase (GST); (**H**) dehydroascorbate reductase (DHAR); (**I**) monodehydroascorbate reductase (MDHAR) of French bean plants grown under control, drought (D), and drought-stressed plants treated with methyl jasmonate (D + MeJA), salicylic acid (D + SA), and their combination (D + MeJA + SA). Vertical bars represent +/− SE values. Different letter(s) denote a significant difference at *p* ≤ 0.05. Additional details are shown in Figure 1.

Drought stress promoted a substantial increase in SOD activity by 54% compared to the unstressed control (Figure 5A and Figure S2A). Exogenous application of MeJA or SA further enhanced SOD activity compared to untreated drought-stressed plants, while the combined MeJA + SA treatment caused a statistically significant 83% rise in SOD activity compared to the untreated drought-stressed plants (Figure S2B). Unlike SOD, the activities of CAT and POD substantially decreased (42% and 54%, respectively) in the drought-stressed plants compared to the controls (Figure 5B,C and Figure S2A). Application of MeJA or SA alone to drought-stressed plants did not show any remarkable effect on these enzyme activities compared to the activities in the untreated drought-stressed plants; however, the combined MeJA + SA treatment significantly increased the activities of CAT and POD (79% and 144%, respectively) compared to activities in the untreated drought-stressed plants (Figure S2B).

A slight increase was noted in APX and GST activities in the drought-stressed plants (36% and 11%, respectively) compared to the unstressed control (Figure 5D,G and Figure S2A). Application of SA alone to drought-stressed plants did not change the APX and GST activities substantially, while MeJA significantly increased the activity of APX only (Figure 5D,G). However, the combined application of MeJA + SA significantly increased the APX and GST activities (73% and 128%) compared to untreated drought-stressed plants (Figure 5D,G and Figure S2B). A marked increase in GPX activity (126%) was recorded in the drought-stressed plants compared to the unstressed controls, but no significant increases were noted for GR activity (Figure 5E,F and Figure S2A). Treatment with MeJA or SA alone did not cause any marked increases, whereas the combined MeJA + SA treatment significantly increased both GPX and GR activities (105% and 134%, respectively) compared to the untreated drought-stressed plants (Figure S2B).

Compared to the unstressed controls, the activities of DHAR and MDHAR decreased (45% and 58%, respectively) in the drought-stressed plants (Figure 5H,I and Figure S2A). Treatment of drought-stressed plants with MeJA or SA alone did not significantly increase these enzyme activities, whereas the combined MeJA + SA treatment resulted in a statistically significant increase in the activities of both DHAR and MDHAR by 59% and 115%, respectively, compared to the untreated drought-stressed plants (Figure 5H,I and Figure S2B). 

### 3.7. Comparative Assessment of Responses across Treatments 

**Figure 6:**
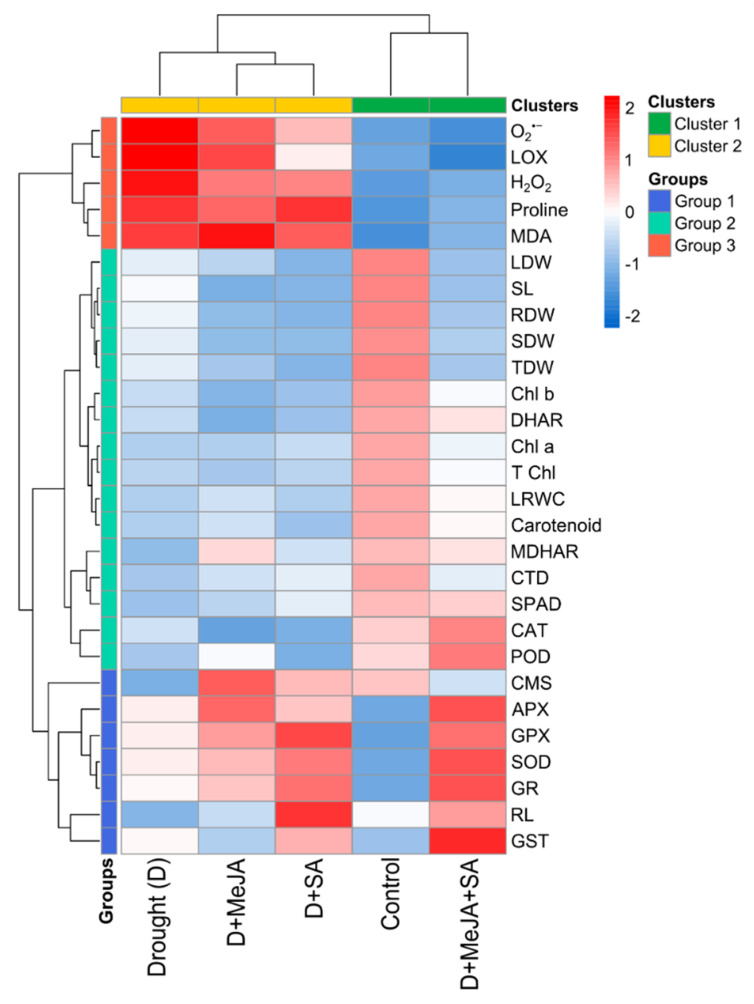
Heatmap and cluster analysis of the growth and physiological attributes, oxidative stress indicators, and antioxidant enzymes in French bean plants grown under control, drought (D), and drought-stressed plants treated with methyl jasmonate (D + MeJA), salicylic acid (D + SA), and their combination (D + MeJA + SA). Additional details are shown in Figure 1.

A comparative heatmap analysis revealed three distinct groups among the parameters measured in this study (Figure 6). Group 1 consisted of most of the antioxidant enzymes (SOD, APX, GPX, GR, and GST), root length, and CMS. Oxidative stress indicators and proline content were placed in group 3, and the rest of the parameters, including CAT, POD, DHAR, and MDHAR, were in group 2. A distinct categorization of the growth, physiological processes, and antioxidant scavenging machinery was observed, as drought-stressed plants without pre-treatment and with pre-treatment with either MeJA or SA alone were grouped in a cluster (Figure 6). However, the plants given the combined MeJA + SA treatment showed significantly higher amelioration of drought stress-induced damage and were grouped in the same cluster as the unstressed control plants (Figure 6). A contrasting response between the unstressed controls and the plants receiving the combined MeJA + SA treatment was observed for the ROS scavenging machinery, whereas the ROS levels and other growth and physiological responses were aligned in the same direction in both plant groups (Figure 6). 

### 3.8. Principal Component Analysis (PCA)

**Figure 7:**
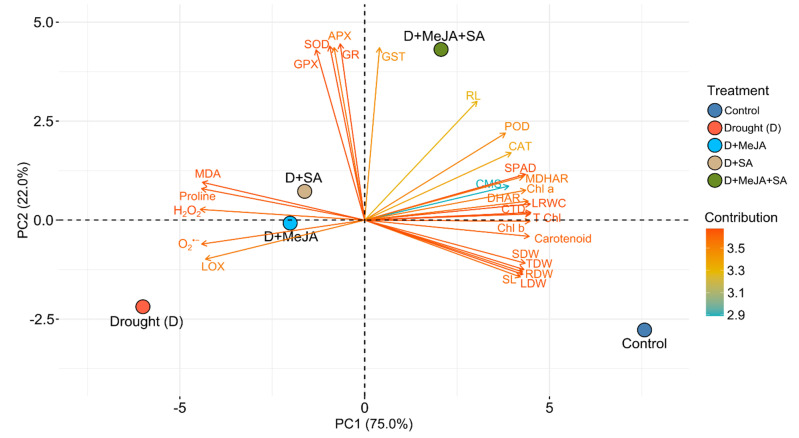
A PCA biplot stipulates the relationship between treatments and observed parameters. Parameters are distributed in different ordinates depending on the divergence among them. In this biplot, the length and color intensity of a vector indicate the quality of representation and the contribution of the traits to the principal components, respectively. The angles between the vectors formed from the middle point of the biplots indicate whether the traits interacted positively or negatively. Additional details are shown in Figure 1.

The PCA of the responses of the French bean plants to different treatments depicted a total of four principal components (PCs), but only two PCs exhibited eigenvalues > 1 and were significant. Both the PCs explained approximately 97% of the variability in drought response (Figure 7 and Table S2). A PCA biplot showed that PC1 exhibited approximately 75% of the total variability and contributed positively via morpho-physiological traits, CAT, POD, DHAR, and MDHAR; it contributed negatively via proline, SOD, H_2_O_2_, MDA, and LOX (Figure 7, Table S2). The second PC accounted for approximately 22% of the total variation and contributed principally by SOD, APX, GPX, GR, GST, and RL and partly by SPAD, CAT, POD, and MDHAR. 

## 4. Discussion 

The findings from this study revealed how exogenous application of MeJA, SA, and their combination can help to reduce the adverse effects of drought in French bean by maintaining cellular water levels, membrane stability, photosynthetic pigment levels, and antioxidant defenses to ultimately improve morpho-physiological growth. The growth parameters of French bean (shoot and root lengths and dry weights) were reduced under drought stress but were less affected following application of MeJA and SA, separately or in combination. An improvement in drought stress tolerance by the combined application of MeJA and SA has recently been demonstrated in maize by Tayyab et al. [27]; however, the present report is the first to demonstrate similar effects in French beans by significant activation of the plant antioxidant defense system. Previous studies have demonstrated an effective minimization of the damaging effects of drought by exogenous application of MeJA or SA alone in mustard [18] and wheat [28] and by the combination of MeJA and SA in maize [27] through enhancement of various biochemical, morphological, and physiological responses [54] including cell elongation, cell expansion, and cell differentiation [55].

The chlorophyll index, including Chl a, b, and total Chl, and the carotenoid content measured in the same leaves were reduced by drought stress (Table 2 and Figure S1A). Drought stress is known to lower the levels of photosynthetic pigments, including chlorophylls and carotenoids, in various crop plants due to the oxidation and impaired biosynthesis of the pigments [56,57]. The observed decrease in chlorophyll concentration under drought stress could represent a suppression of chlorophyll biosynthesis and/or decreased synthesis and assembly of the PSI and PSII light-harvesting complexes to suppress excess absorption and ROS production [6,58]. The negative impacts on pigments were partly ameliorated in French bean plants treated with MeJA or SA, but the effects were consistently statistically significant for the combined MeJA + SA treatment, and the plant growth and physiology were similar to those of the unstressed control plants (Table 2 and Figure S1B). Increases in leaf chlorophyll and carotenoid contents in drought-stressed plants treated with MeJA, SA, and their combination have been reported previously in soybean [59], soybean [60], and maize [27], respectively.

Canopy temperature depression, LRWC, and CMS are considered effective indicators of drought stress tolerance [6,61,62,63]. Our findings provide support for earlier results showing that French beans under drought stress have significantly lower CTD, LRWC, and CMS; however, these reductions were suppressed by the application of MeJA or SA and statistically significantly suppressed by the combined MeJA + SA treatment (Figure 3 and Figure S1B). CTD is regarded as a reliable indication of plant water status, and a positive CTD means the canopy is cooler than the surrounding air [64]. 

An improved LRWC was reported in drought-stressed plants following MeJA treatment of soybean [59], SA treatment of soybean [60] and *Ctenanthe setosa* [65], and a combined MeJA and SA treatment of maize [27]. The enhanced CTD, LRWC, and CMS in the drought-stressed French bean plants treated with MeJA and SA in the present study indicates that these hormones, particularly when supplied in combination, have the potential to reduce the harmful effects of drought by maintaining cellular water status and maintaining a cooler leaf temperature.

Proline has been considered an osmotic stress mediator, stabilizer of macromolecules, compatible solutes to preserve enzymes, and capable of storing carbon and nitrogen for usage during drought and other stresses [66]. Proline accumulation in plants is also a marker of stress induction [67]. Although proline content profoundly increased under drought stress in French beans, the MeJA, SA, and MeJA + SA treatments markedly reduced the proline content in drought-stressed plants (Figure 3E and Figure S1B). This was due to the reduction in osmotic stress following the application of hormones [18,24,26].

The effect of exogenous MeJA and SA on the drought-induced oxidative stress was investigated by determining O_2_^●−^ generation and the H_2_O_2_ and MDA levels as well as the specific activity of LOX. Drought stress significantly increased the levels of O_2_^●−^, H_2_O_2_, and MDA and LOX activity compared to the unstressed control plants, indicating that the plants were under severe oxidative stress (Figure 4 and Figure S2A). The drought-induced increases in oxidative stress indicators in our study were consistent with results from previous studies on soybean [60], mustard [24], and maize [27]. Exogenous application of MeJA or SA suppressed the production of the oxidative stressors, while MeJA + SA showed statistically significant effects in drought-stressed French bean plants (Figure 4 and Figure S2B). These findings are in accordance with previous studies showing that combined treatments with MeJA and SA reduced the levels of oxidative stress indicators in maize seedlings under drought [27] and in Eureka lemon under chilling stress [30].

In plant cells, SOD renders key protection against O_2_^•^^−^ by converting it to H_2_O_2_ and, subsequently, neutralizing it to H_2_O by CAT and peroxidases (POD, APX, and GPX) [15,16,68]. The plant GSTs are a large and diverse group of enzymes that catalyze the coupling of electrophilic xenobiotic substrates with GSH and are linked with the induction of tolerance to different abiotic stresses [69]. The four enzymes of the ascorbate–glutathione cycle (i.e., APX, MDHAR, DHAR, and GR) played a pivotal role in the systematic detoxification of cellular H_2_O_2_ produced due to the oxidative stress in wheat [41].

In the present study, increased activity of SOD, APX, GPX, GST, and GR and decreased activity of CAT, POD, DHAR, and MDHAR were observed in drought-stressed plants compared with unstressed the control plants (Figure 5), which is in agreement with earlier findings of similar drought-stress effects in rapeseed [70], mustard [18], and mung bean [71]. Exogenous MeJA, SA, and their combination augmented the antioxidant enzyme activities in French beans under drought stress and reduced drought-induced oxidative damage (Figure 5 and Figure S2B). However, MeJA (20 μM) and SA (2 mM) applied independently did not consistently improve the antioxidant enzyme system under drought; this may be because of a reliance on other stress signals that can activate antioxidant enzymes’ activities [55]. MeJA or SA induced an upregulation of the antioxidant enzyme system under drought stress in *Ctenanthe setosa* [65], *Cucumis melo* [72], mustard [18,24], jatropha [22], and *Verbascum sinuatum* [26]. However, a combined MeJA and SA treatment (10 μM + 1 mM) was more effective in this upregulation of the antioxidant enzymes (Figure 5 and Figure S2B). These results are in agreement with Tayyab et al. [27], who observed a synergistically positive role of MeJA + SA in increasing antioxidant enzyme activity under drought-induced oxidative stress in maize seedlings. Our results suggest that the signaling routes of MeJA and SA would operate similarly to trigger defensive responses in French bean plants under drought stress. However, investigating additional mechanisms that lead to an increased effectiveness by combined hormone applications on minimizing drought stress is an intriguing area for future research.

Taken together, our results demonstrated that MeJA or SA reduced oxidative stress by upregulating antioxidant enzyme activities, improving the physiological functions of French bean plants under drought stress; however, the alleviation was further boosted by combined hormone application. Heatmap and PCA biplot analyses clearly showed that the combined application of MeJA and SA mitigated drought-induced oxidative stress principally by the upregulation of SOD, APX, GPX, GR, and GST and partly by CAT, POD, and MDHAR activities (Figure 6 and Figure 7). The results presented in this study add new insights into oxidative stress defense mechanisms triggered by MeJA and SA in French bean plants.

## 5. Conclusions

French beans pre-treated with exogenous MeJA and SA showed reductions in drought stress responses including improved photosynthetic performance, membrane stability, water status, leaf temperature control, and antioxidant enzyme activities. Interestingly, the combined application of MeJA (10 μM) and SA (1 mM) was more efficacious at alleviating the adverse effects of drought-induced oxidative stress by upregulating antioxidant enzymatic activities. The combined treatment also improved the physiological activities and increased plant biomass compared to drought-stressed plants. Seed and foliar treatment of French bean with a combination of MeJA and SA significantly improved drought stress tolerance by augmenting antioxidant systems. The findings from this study provide the rationale for future research related to the defense signaling pathways employed by French bean plants pre-treated with MeJA and SA to aid in the development of new cultivars with improved adaptation to future drier scenarios.

## Figures and Tables

**Table 1:** Effect of methyl jasmonate (MeJA), salicylic acid (SA), and their combined application on the growth parameters of French beans under drought stress.

Treatment	Root Length (cm)	Shoot Length (cm)	Root Dry Weight (g)	Shoot Dry Weight (g)	Leaf Dry Weight (g)	Total Dry Weight (g)
Control	58.63 ± 3.45 ^a^	36.49 ± 0.87 ^a^	2.88 ± 0.26 ^a^	9.04 ± 0.74 ^a^	9.93 ± 0.77 ^a^	21.86 ± 1.62 ^a^
Drought (D)	55.69 ± 1.23 ^a^	21.27 ± 0.65 ^c^	1.38 ± 0.12 ^b^	2.77 ± 0.21 ^c^	2.61 ± 0.28 ^d^	6.76 ± 0.35 ^c^
D + MeJA	57.59 ± 2.13 ^a^	22.10 ± 0.49 ^c^	1.53 ± 0.17 ^b^	3.55 ± 0.19 ^c^	3.91 ± 0.37 ^b c^	8.99 ± 0.64 ^c^
D + SA	58.91 ± 2.55 ^a^	22.46 ± 0.63 ^c^	1.54 ± 0.18 ^b^	3.69 ± 0.49 ^c^	3.62 ± 0.13 ^c d^	8.85 ± 0.65 ^c^
D + MeJA + SA	59.81 ± 1.07 ^a^	25.63 ± 0.55 ^b^	1.86 ± 0.19 ^b^	5.09 ± 0.28 ^b^	4.89 ± 0.12 ^b^	11.84 ± 0.23 ^b^

Values represent the mean ± SE. Values in a column with distinct letter(s) were significantly different at *p* ≤ 0.05. Control—plants grown under non-stress, well-irrigated conditions; drought—plants grown with a steady decline in moisture availability; D + MeJA—drought-stressed plants pre-treated (seed and foliar) with 20 µM methyl jasmonate; D + SA—drought-stressed plants pre-treated (seed and foliar) with 2 mM salicylic acid; D + MeJA + SA—drought-stressed plants pre-treated (seed and foliar) with a combination of 10 µM methyl jasmonate and 1 mM salicylic acid.

**Table 2:** Effect of methyl jasmonate (MeJA), salicylic acid (SA), and their combination on SPAD value and leaf pigment contents of French bean plants under drought stress.

Treatments	SPAD	Leaf Pigments (mg g^−1^ Fresh Weight)			
		Chl a	Chl b	Total Chl	Carotenoids
Control	49.85 ± 0.49 ^a^	1.39 ± 0.03 ^a^	0.49 ± 0.02 ^a^	1.97 ± 0.06 ^a^	0.50 ± 0.03 ^a^
Drought (D)	39.65 ± 1.18 ^c^	0.65 ± 0.10 ^c^	0.24 ± 0.05 ^c^	0.98 ± 0.07 ^c^	0.26 ± 0.01 ^c^
D + MeJA	43.65 ± 0.93 ^b^	0.87 ± 0.11 ^bc^	0.29 ± 0.04 ^bc^	1.25 ± 0.17 ^bc^	0.34 ± 0.05 ^bc^
D + SA	44.35 ± 1.52 ^b^	0.91 ± 0.16 ^bc^	0.30 ± 0.07 ^bc^	1.30 ± 0.25 ^bc^	0.33 ± 0.06 ^bc^
D + MeJA + SA	48.18 ± 0.94 ^a^	1.12 ± 0.06 ^b^	0.39 ± 0.02 ^ab^	1.61 ± 0.08 ^ab^	0.42 ± 0.03 ^ab^

Values represent the mean ± SE. Values in a column with distinct letter(s) were significantly different at *p* ≤ 0.05. Additional details are listed in Table 1.

## Data Availability

All relevant data are available in this manuscript.

## Supplementary Materials

The following are available online at https://www.mdpi.com/article/10.3390/plants10102066/s1, Table S1: Daily weather data at the experimental site during the study period, Table S2: Extracted Eigenvalues and latent vectors of studied traits associated with the first two principal components, Figure S1: Variations in the studied growth and physiological parameters of French bean plants. A. Percent change due to drought over control and B. percent change over drought due to the application of methyl jasmonate (D + MeJA), salicylic acid (D + SA) and their combination (D + MeJA + SA) on drought-stressed plants, and Figure S2: Variations in the oxidative stress indicators and antioxidant enzymes activity of French bean plants. A. Percent change due to drought over control and B. percent change over drought due to the application of methyl jasmonate (D + MeJA), salicylic acid (D + SA) and their combination (D + MeJA + SA) on drought stressed plants.
