# Supplementary material for: Exogenous Application of Methyl Jasmonate and Salicylic Acid Mitigates Drought-Induced Oxidative Damages in French Bean (Phaseolus vulgaris L.)
Source: Plants (Basel). 2021 Sep 30;10(10):2066. doi: 10.3390/plants10102066 (PMC8538183; doi:10.3390/plants10102066)
Supplement: Supplementary file 1 [file plants-10-02066-s001.zip › plants-1376817-supplementary.pdf]

Table S1. Daily weather data at the experimental site during the study period

Date	Air Temperature (°C)			Humidity (%)	Rainfall (mm)	Actual Evaporation (mm)
	Max.	Min.	Ave.			
5 Dec 2020	28.0	14.5	21.3	82	0	1.5
6 Dec 2020	27.0	15.0	21.0	91	0	1.33
7 Dec 2020	27.5	16.0	21.8	90	0	0.97
8 Dec 2020	27.0	17.0	22.0	90	0	0.88
9 Dec 2020	25.5	18.0	21.8	90	0	0.93
10 Dec 2020	20.0	15.0	17.5	90	0	0.44
11 Dec 2020	22.0	18.0	20.0	90	0	0.53
12 Dec 2020	20.0	14.5	17.3	90	0	0.44
13 Dec 2020	23.0	14.5	18.8	90	0	0.71
14 Dec 2020	25.0	14.0	19.5	91	0	0.84
15 Dec 2020	26.5	15.0	20.8	89	0	1.15
16 Dec 2020	21.5	15.0	18.3	90	0	0.53
17 Dec 2020	26.5	14.0	20.3	81	0	1.06
18 Dec 2020	26.0	12.0	19.0	81	0	1.11
19 Dec 2020	24.0	8.0	16.0	82	0	0.97
20 Dec 2020	24.0	11.0	17.5	79	0	1.15
21 Dec 2020	24.5	10.5	17.5	89	0	1.2
22 Dec 2020	25.0	10.0	17.5	90	0	1.33
23 Dec 2020	26.0	11.0	18.5	90	0	1.42
24 Dec 2020	26.5	11.5	19.0	90	0	1.46
25 Dec 2020	26.0	11.0	18.5	90	0	1.02
26 Dec 2020	27.0	12.0	19.5	80	0	1.24
27 Dec 2020	26.0	14.5	20.3	80	0	1.06
28 Dec 2020	25.0	11.0	18.0	80	0	0.97
29 Dec 2020	25.5	10.5	18.0	80	0	0.88
30 Dec 2020	26.0	12.0	19.0	90	0	1.06
31 Dec 2020	27.0	11.5	19.3	90	0	1.15
1 Jan 2021	27.0	11.0	19.0	90	0	1.11
2 Jan 2021	27.5	11.5	19.5	90	0	1.15
3 Jan 2021	27.0	11.0	19.0	81	0	1.24
4 Jan 2021	28.0	12.0	20.0	90	0	1.28
5 Jan 2021	27.5	12.0	19.8	81	0	1.33

Date	Air Temperature (°C)			Humidity (%)	Rainfall (mm)	Actual Evaporation (mm)
	Max.	Min.	Ave.			
6 Jan 2021	28.0	13.5	20.8	81	0	1.5
7 Jan 2021	30.0	15.0	22.5	91	0	1.68
8 Jan 2021	30.0	14.5	22.3	81	0	1.77
9 Jan 2021	29.5	14.0	21.8	90	0	1.42
10 Jan 2021	27.5	15.0	21.3	81	0	1.28
11 Jan 2021	27.0	15.0	21.0	91	0	0.97
12 Jan 2021	28.0	15.0	21.5	90	0	1.06
13 Jan 2021	26.0	11.0	18.5	80	0	0.93
14 Jan 2021	23.0	13.0	18.0	90	0	0.88
15 Jan 2021	24.0	10.0	17.0	89	0	1.06
16 Jan 2021	24.0	11.0	17.5	90	0	1.15
17 Jan 2021	23.5	10.0	16.8	88	0	1.06
18 Jan 2021	23.0	11.0	17.0	88	0	0.88
19 Jan 2021	23.5	10.0	16.8	89	0	0.97
20 Jan 2021	24.0	13.5	18.8	90	0	1.11
21 Jan 2021	22.0	14.0	18.0	90	0	1.68
22 Jan 2021	24.0	13.0	18.5	89	0	0.97

Source: Weather Station, Department of Agricultural Engineering, Bangabandhu Sheikh Mujibur Rahman Agricultural University, Gazipur, Bangladesh.

Table S2. Extracted Eigenvalues and latent vectors of studied traits associated with the first two principal components.

Variable	Principal components	
	PC1	PC2
Extracted Eigenvalues	21.736	6.379
Explained variance (%)	75.0	22.0
Cumulative variance (%)	75.0	97.0
	Latent vectors	
RL	0.689	0.657
SL	0.926	-0.342
RDW	0.941	-0.313
SDW	0.957	-0.264
LDW	0.944	-0.325
TDW	0.950	-0.299
CMS	0.877	0.183
LRWC	0.997	-0.070
CTD	0.997	0.016
SPAD	0.971	0.237
Chl a	0.999	0.025
Chl b	0.991	-0.029
T Chl	0.998	0.021
Carotenoid	0.996	0.045
Proline	-0.977	0.197
H_2O_2	-0.988	0.077
MDA	-0.967	0.234
$O_2^{\bullet-}$	-0.984	-0.117
LOX	-0.962	-0.203
SOD	-0.191	0.979
CAT	0.884	0.353
POD	0.855	0.468
APX	-0.163	0.971
GPX	-0.272	0.961
GR	-0.127	0.990
GST	0.105	0.959
DHAR	0.982	0.082
MDHAR	0.962	0.228

APX− ascorbate peroxidase, CAT− catalase, Chl a− chlorophyll a, Chl b− chlorophyll b, CMS− cell membrane stability, CTD− canopy temperature depression, DHAR− dehydroascorbate reductase, GPX− glutathione peroxidase, GR− glutathione reductase, GST− glutathione-S-transferase, H_2O_2− hydrogen peroxide, LDW− leaf dry weight, LOX− lipoxygenase, LRWC−leaf relative water content, MDA− malondialdehyde, MDHAR− monodehydroascorbate reductase, $O_2^{\bullet-}$− superoxide radical, POD− guaiacol

peroxidases, RDW− root dry weight, RL− root length, SDW− shoot dry weight, SL− shoot length, SOD−superoxide dismutase, SPAD− chlorophyll index, TDW− total dry weight, and T Chl− total chlorophyll.

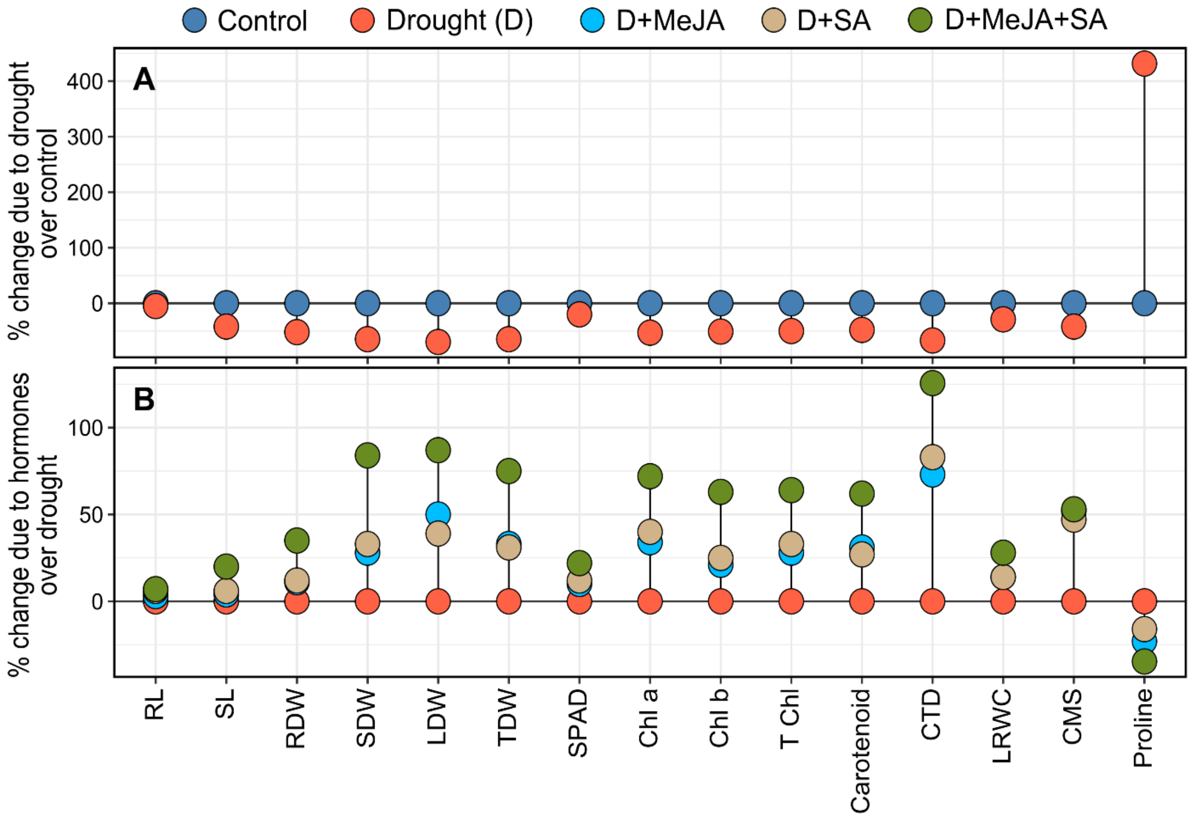

Figure S1. Variations in the studied growth and physiological parameters of French bean plants. A. Percent change due to drought over control and B. percent change over drought due to the application of methyl jasmonate (D+MeJA), salicylic acid (D+SA) and their combination (D+MeJA+SA) on drought-stressed plants. Control– plants grown under non-stress well-irrigated conditions, Drought– plants grown with a steady decline in moisture availability, D+MeJA– drought + seed and foliar pre-treatment with 20 μM methyl jasmonate, D+SA– drought + seed and foliar pre-treatment with 2 mM salicylic acid, and D+MeJA+SA– drought + seed and foliar pre-treatment with combination of 10 μM methyl jasmonate and 1 mM salicylic acid.

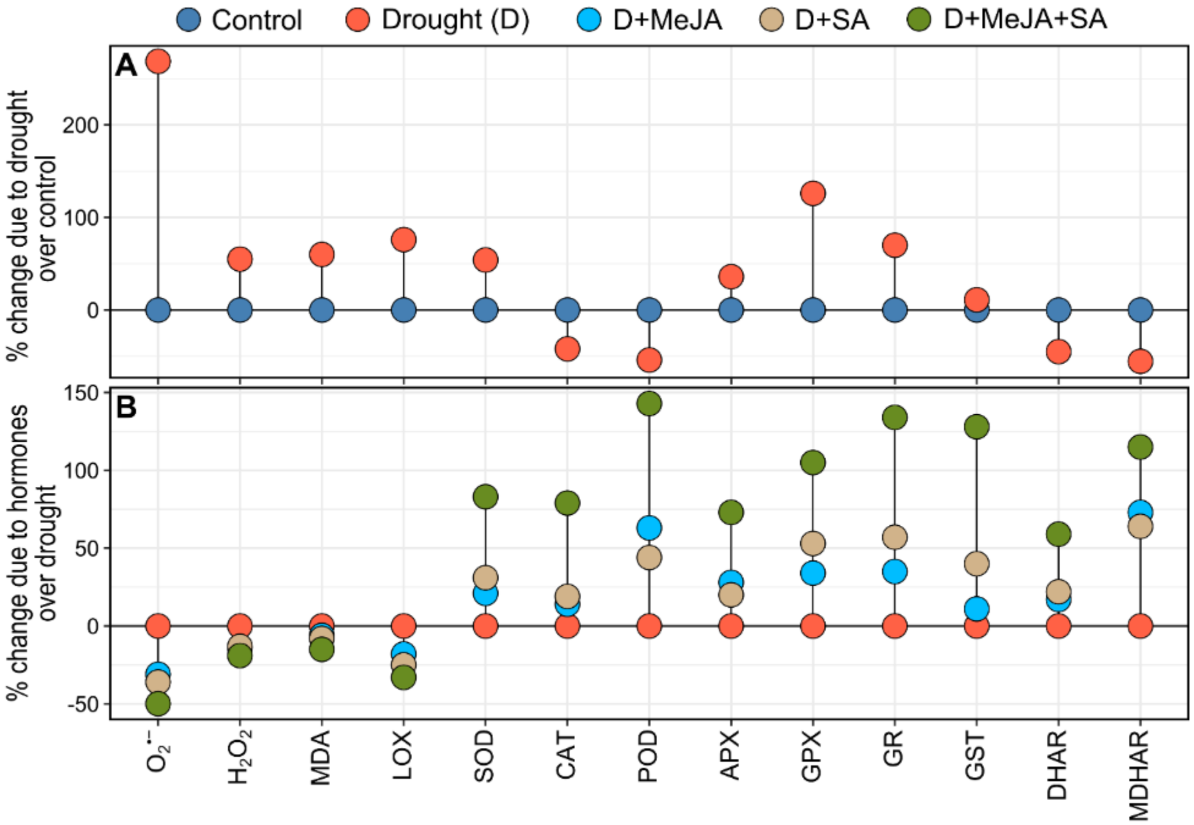

Figure S2. Variations in the oxidative stress indicators and antioxidant enzymes activity of French bean plants. A. Percent change due to drought over control and B. percent change over drought due to the application of methyl jasmonate (D+MeJA), salicylic acid (D+SA) and their combination (D+MeJA+SA) on drought stressed plants. Control− plants grown under non-stress well-irrigated conditions, Drought− plants grown with a steady decline in moisture availability, D+MeJA− drought + seed and foliar pre-treatment with 20 µM methyl jasmonate, D+SA− drought + seed and foliar pre-treatment with 2 mM salicylic acid, and D+MeJA+SA− drought + seed and foliar pre-treatment with combination of 10 µM methyl jasmonate and 1 mM salicylic acid.
